# Shear Testing of the Interfacial Friction Between an HDPE Geomembrane and Solid Waste

**DOI:** 10.3390/ma13071672

**Published:** 2020-04-03

**Authors:** Luming Zhou, Zhende Zhu, Zhenpeng Yu, Cong Zhang

**Affiliations:** 1 Key Laboratory of Ministry of Education for Geomechanics and Embankment Engineering, Hohai University, Nanjing 210098, China; sk_zlm@163.com (L.Z.); hhzzde@163.com (Z.Z.); 2 Jiangsu Research Center for Geotechnical Engineering Technology, Hohai University Nanjing 210098, China; 3 School of Transportation, Southeast University, Nanjing Hydraulic Research Institute, Nanjing 211102, China; dnyuzp@163.com

**Keywords:** HDPE geomembrane, solid waste, interfacial friction, shear strength, direct shear test

## Abstract

High-density polyethylene (HDPE) geomembrane is often used as an anti-seepage material in domestic and industrial solid waste landfills. To study the interfacial shear strength between the HDPE anti-seepage geomembrane and various solid wastes, we performed direct shear tests on the contact interface between nine types of industrial solid waste or soil (desulfurization gypsum, fly ash, red mud, mercury slag, lead-zinc slag, manganese slag, silica fume, clay and sand) and a geomembrane with a smooth or rough surface in Guizhou Province, China. Friction strength parameters like the interfacial friction angle and the apparent cohesion between the HDPE geomembrane and various solid wastes were measured to analyze the shear strength of the interface between a geomembrane with either a smooth or a rough surface and various solid wastes. The interfacial shear stress between the HDPE geomembrane and the industrial solid waste increased with shear displacement and the slope of the stress-displacement curve decreased gradually. When shear displacement increased to a certain range, the shear stress at the interface remained unchanged. The interfacial shear strength between the geomembrane with a rough surface and the solid waste was higher than for the geomembrane with a smooth surface. Consequentially, the interfacial friction angle for the geomembrane with a rough surface was larger. The geomembrane with a rough surface had a better shear resistance and the shear characteristics fully developed when it was in full contact with the solid waste.

## 1. Introduction

A high-density polyethylene (HDPE) geomembrane is a waterproof barrier material with a high strength made of a polyethylene resin with a specific formula and further processing [1]. It is widely used for the anti-seepage engineering of various domestic and solid waste residues because of its good resistance to low temperatures and good corrosion resistance [2,3,4,5,6]. The friction of the interface between the HDPE geomembrane and the solid waste under direct shear must be well considered to assess the stability of engineering structures [7,8,9,10,11]. According to the standard for pollution control on the storage and disposal of general industrial solid waste in the Guizhou province, the yard storing of slag from class II general industrial solid waste must use anti-seepage methods [12]. HDPE geomembranes or composite structures containing an HDPE geomembrane were generally adopted for the anti-seepage structure. The HDPE geomembrane is a ductile material in contact with solid waste, clay, concrete or sand on both sides and most of the geomembrane is laid on a slope [13,14,15,16]. Due to the settlement and deformation of the stock filler, the HDPE geomembrane is under shear tension [17,18,19,20,21]. Taking measures to ensure that the geomembrane will not be damaged or slide during its operation is important for solid waste disposal areas located in valleys.

Presently, the experimental researches on the interfacial strength between the HDPE geomembrane and various types of solid waste mainly focus on direct shear, drawing and triaxial or torsional shear. Ling et al. [22] measured the friction strength of the contact interface between a geomembrane and clay with a 100 mm × 100 mm direct shear apparatus and analyzed the relationship between the shear displacement and the maximum shear stress at the contact interface. Fox et al. [23] studied the shear failure characteristic of the contact interface between a gravel foundation and a 1.5 mm thick HDPE and a low-density polyethylene (LDPE) geomembrane both with a smooth surface under different shear loads. Lin et al. [24] analyzed the transfer mechanism of the shear force in a geomembrane liner using sandbag loads to simulate the landfills. Gao et al. [25] studied the interfacial strength between a reinforced HDPE geomembrane and sand under a different normal stress through a large number of direct shear tests. However, at present, most of the researches on the shear strength of the interface between geomembrane and soil are conducted with sand or clay, because these two kinds of soil samples are common and easy to obtain, while few scholars study the shear strength of the interface between different industrial solid wastes and geomembrane, this is because industrial solid wastes are difficult to obtain and have certain toxicity. Few studies on the stress and strain of an anti-seepage geomembrane used in various solid waste disposal sites and different operation conditions. The design and construction of the anti-seepage system of each waste disposal site only relies on the experience of the individuals involved, which does not assure the safety of the anti-seepage system of the waste disposal site. Therefore, it is necessary to carry out more specific and systematic research [26,27,28,29,30,31,32,33].

In this study, we used a modified direct shear apparatus to carry out direct shear tests on smooth and rough HDPE geomembranes and nine types of industrial solid waste or soil to simulate their interaction in an actual engineering structure and measure the friction strength parameters like the interfacial friction angle and the apparent cohesion of the contact interface between both types of geomembrane and the solid waste. We selected enough types (nine kinds) of industrial solid wastes or soil samples to verify that the conclusions obtained are applicable to the vast majority of industrial solid wastes. At the same time, we used the modified fracture shear seepage coupling system to carry out the test, which could keep the shear area of the interface between geomembrane and solid wastes unchanged during the test and the data of the stress and strain could be recorded per second accurately. The shear characteristics of the contact interface between both types of HDPE geomembrane and various industrial solid waste or soil samples were evaluated and analyzed to provide a theoretical reference for the construction of anti-seepage systems in solid waste disposal sites.

## 2. Materials and Methods

### 2.1. Test Equipment

**Figure 1:**
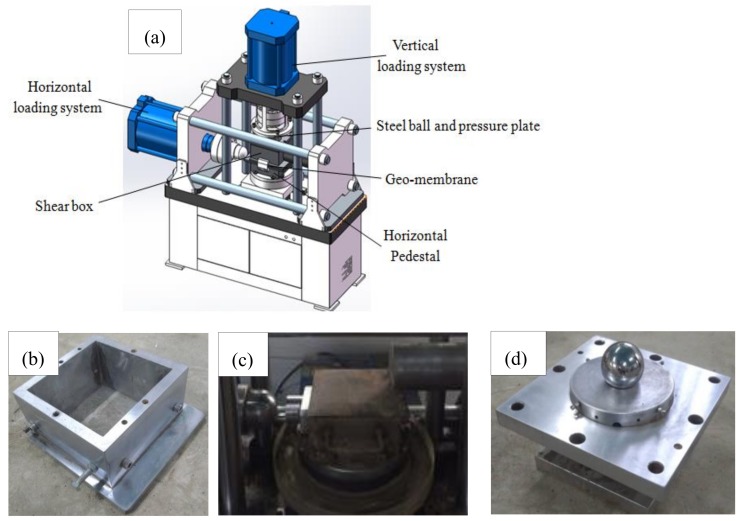
Test equipment: (**a**) schematic view of fracture shear-seepage coupling test system; (**b**) shear box; (**c**) horizontal pedestal; (**d**) steel ball and pressure plate.

Figure 1a shows the fracture shear seepage coupling system, developed by the Nanjing Hydraulic Research Institute (Nanjing, China) and Xi’an Lichuang Material Testing Technology Co. Ltd. (Xi’an, China), used in this work. The effective size of the upper shear box (Figure 1b) was 210 × 210 × 130 mm. The original lower shear box was modified into a solid rigid cube with an effective size of 250 × 250 × 10 mm and used as a horizontal pedestal (Figure 1c). The long side of the horizontal pedestal was 40 mm larger than the effective size of the upper shear box, which ensured that the shear area remained unchanged during the test. The maximum static load of the horizontal loading system of the instrument could reach 1000 kN and the stroke of the actuator reached 100 mm. The load applied by the vertical loading system was transferred to the soil through the steel ball and the pressure plate (Figure 1d). The ball head structure could effectively compensate the bias load of the test force caused by the shape and the error in the positioning of the specimen. This made sure that the deformation center in the x-direction coincided with the center of the specimen. The maximum static load of the vertical loading system of the instrument could reach 500 kN and the stroke of the actuator reached 100 mm. The load sensor was imported from the United States. The displacement sensor was a high-precision magnetostrictive sensor and used a linear variable differential transformer electromechanical to provide an accurate measurement and transmission of the load and the displacement values.

### 2.2. Test Materials

#### 2.2.1. HDPE Geomembrane

**Figure 2:**
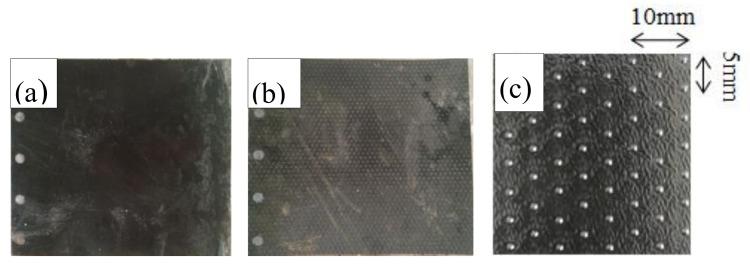
Physical aspect of the high-density polyethylene (HDPE) geomembrane with a (**a**) smooth and (**b**) rough surface; (**c**) the arrangement of bulges on the surface of rough geomembrane.

Figure 2a,b shows both types of HDPE geomembrane with a thickness of 2.5 mm (smooth and rough) produced by Changsha Jianyi New Material Co., Ltd. (Changsha, China), selected for the test. Figure 2c shows the arrangement of bulges on the surface of rough geomembrane. The bulge of the rough geomembrane is hemispherical and arranged in quincunx shape. The height of the bulge is 0.25 mm and the diameter of the bulge is 1.5 mm. Table 1 shows the physical properties of the HDPE geomembrane (minimum).

#### 2.2.2. Filled Soil

**Figure 3:**
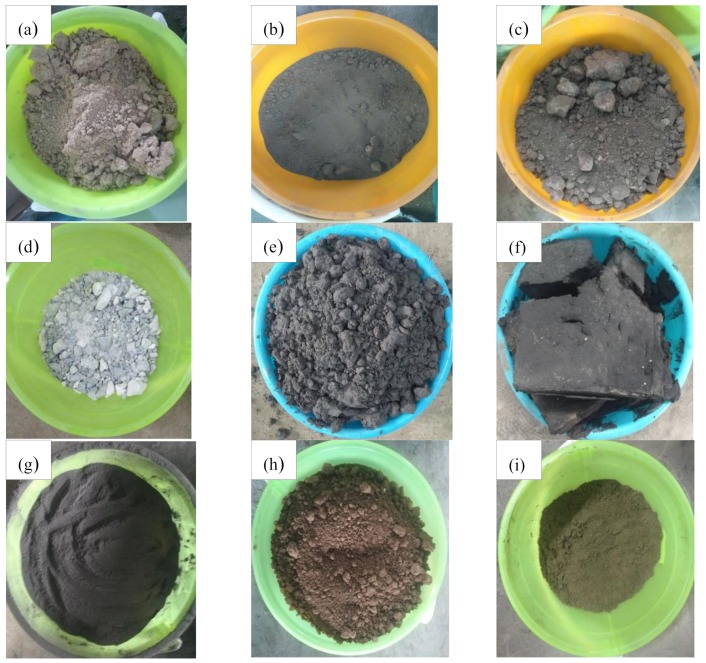
Physical aspect of each filler. (**a**) desulfurized gypsum; (**b**) fly ash; (**c**) red mud; (**d**) mercury slag; (**e**) lead-zinc slag; (**f**) manganese slag; (**g**) silica fume; (**h**) clay and (**i**) sand.

Figure 3a–i shows the seven types of main industrial solid waste, namely desulfurized gypsum, fly ash, red mud, mercury slag, lead-zinc slag, manganese slag and silica fume, as well as both types of soil, namely clay and sand from the Guizhou Province, that were selected.

#### 2.2.3. Test Method

According to the coulombic shear criterion, four geomembrane specimens were used to measure the shear stress when the contact interface between the geomembrane and the soil was damaged under four levels of vertical load. Then, the friction angle and the apparent cohesion of the contact interface between the geomembrane and the soil were determined. Based on the relevant specifications [34], specific steps were defined:

(1) The smooth (or rough) geomembrane was laid on a rigid horizontal base in the lower part of the shear box. The front end was clamped in front of the shear area and fixed in place with 4 bolts. The surface of the geomembrane remained flat without any folding and there was no relative sliding between the specimen and the base. Then, the shear box was installed, and the solid waste was used as filler. The solid waste was filled into the shear box and the contact surface between the solid waste and the geomembrane and the upper surface of the solid waste were kept flat.
(2) The pressure plate was installed, and the solid waste was applied a normal pressure of 50 kPa.
(3) The horizontal load was applied to obtain a relative displacement between the upper and lower shear boxes with a speed of 1 mm/min. The instrument automatically recorded the shear force and the corresponding relative displacement at an interval of 1 s until the horizontal load did not increase anymore, which meant that the geomembrane was sheared out. If the horizontal load kept increasing slowly, the test was carried out until 16.5% of the length of the shear plane was reached.
(4) The geomembrane and the solid waste were removed from the geomembrane surface. It was inspected whether the geomembrane was elongated, folded or damaged.
(5) Re-assembled the geomembrane and repeated steps (1)–(4) and measured the friction characteristics of the contact interface for three other normal pressure values (100 kPa, 150 kPa and 200 kPa).

#### 2.2.4. Stress Calculation

We calculated the shear displacement and the shear stress of the contact surface between the geomembrane and the solid waste according to Equations (1) and (2):

(1) $$\Delta L=L_{t}-L_{0}$$

(2) $$\tau =\frac{T_{t}-T_{0}}{A}$$

where ΔL is the shear displacement at a time t, in mm, $L_{t}$ is the horizontal displacement of the geomembrane at a time t, in mm, $L_{0}$ is the initial horizontal displacement, in mm, τ is the shear stress of the contact surface, in kPa, $T_{t}$ is the horizontal load at a time t, in kN, $T_{0}$ is the initial horizontal load, in kN and A was the contact area, (0.04 m^2^).

## 3. Results and Analysis

### 3.1. Relationship between the Shear Stress and the Shear Displacement

**Figure 4:**
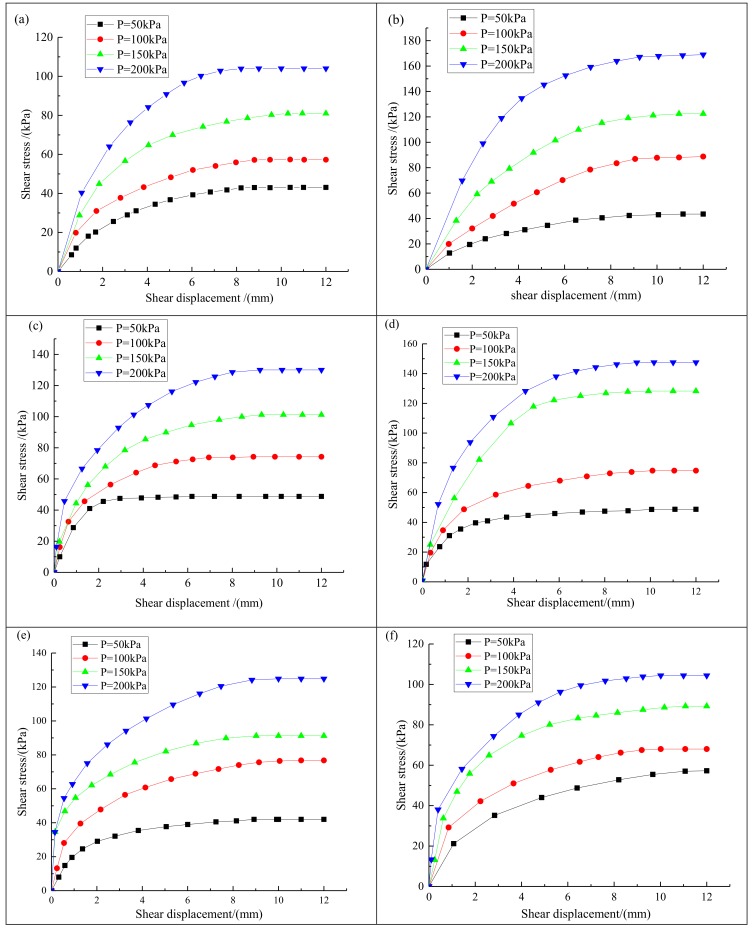

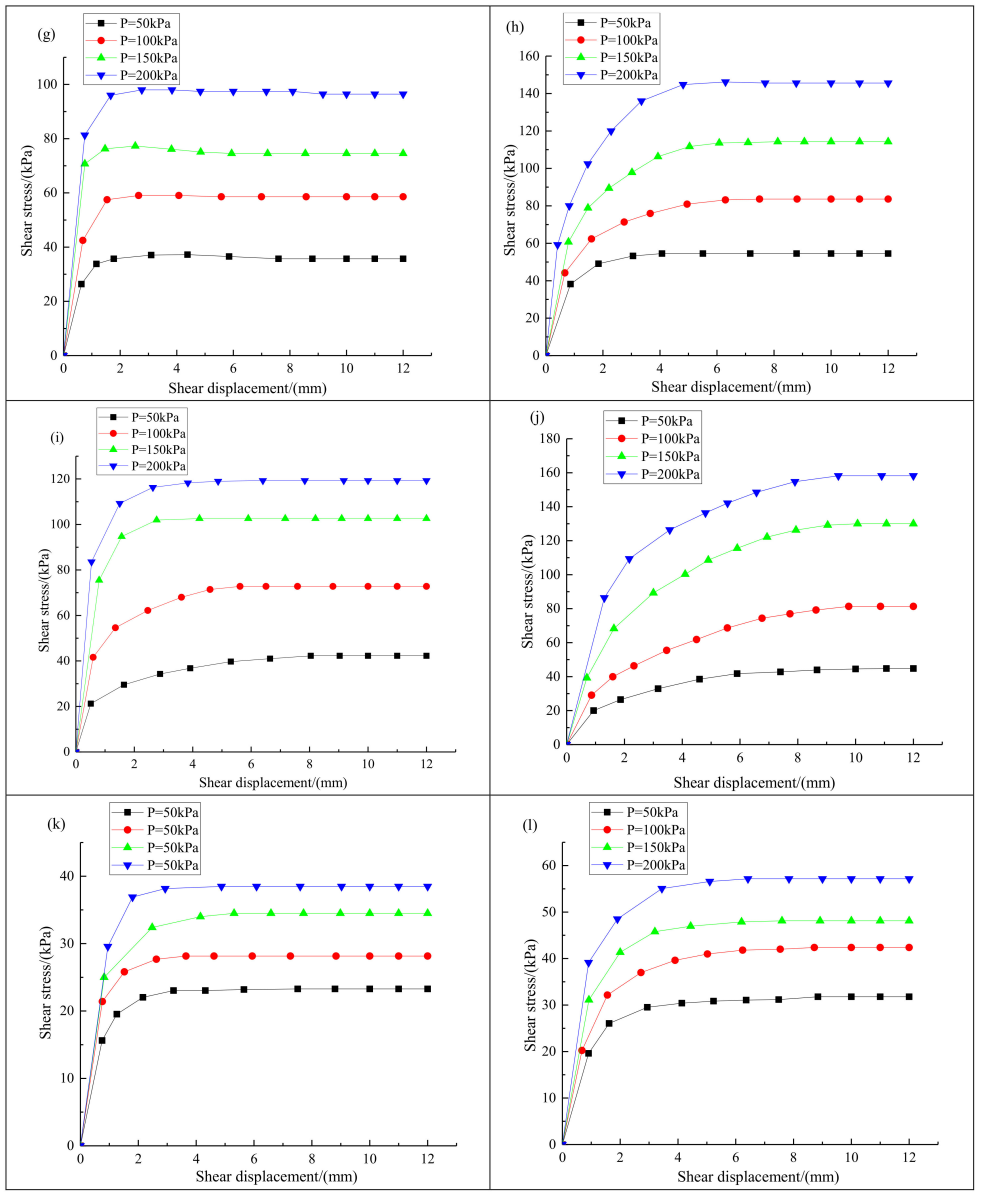

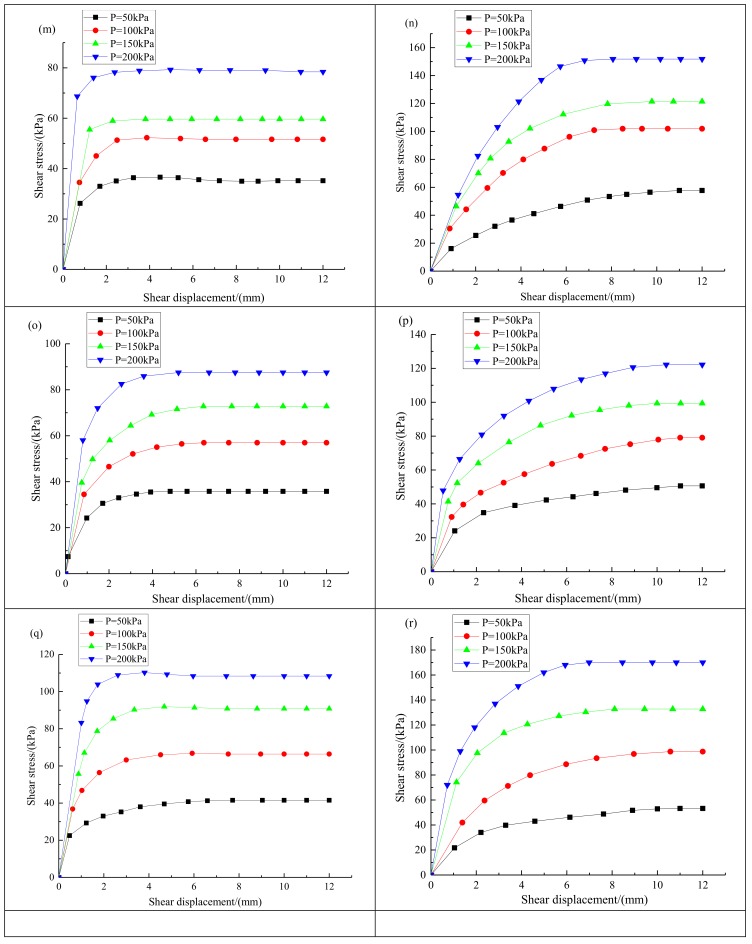
Relationship curve between the shear stress and the shear displacement for the geomembrane and different solid wastes and soils: (**a**) geomembrane with smooth surface and desulfurized gypsum; (**b**) geomembrane with rough surface and desulfurized gypsum; (**c**) geomembrane with smooth surface and fly ash; (**d**) geomembrane with rough surface and fly ash; (**e**) geomembrane with smooth surface and red mud; (**f**) geomembrane with rough surface and red mud; (**g**) geomembrane with smooth surface and mercury slag; (**h**) geomembrane with rough surface and mercury slag; (**i**) geomembrane with smooth surface and lead–zinc slag; (**j**) geomembrane with rough surface and lead-zinc slag; (**k**) geomembrane with smooth surface and manganese slag; (**l**) geomembrane with rough surface and manganese slag; (**m**) geomembrane with smooth surface and silica fume; (**n**) geomembrane with rough surface and silica fume; (**o**) geomembrane with smooth surface and clay; (**p**) geomembrane with rough surface and clay; (**q**) geomembrane with smooth surface and sand; (**r**) geomembrane with rough surface and sand.

The evolution of the shear stress with the shear displacement was plotted systematically. The peak value of the shear stress on the curve was defined as the maximum shear stress. Figure 4a–r shows the relationship between the shear stress and the shear displacement for the geomembranes with a smooth and a rough surface and different solid wastes and soils.

Figure 4a–r shows that the change trend of the shear stress–shear displacement curve for the contact interface between geomembrane and various industrial solid wastes was relatively similar. Each curve was approximately parabolic and their slopes decreased gradually under the action of the four normal loads. Taking desulfurized gypsum as an example, there were two stages in the evolution of the shear stress. First, for a shear displacement within 1–3 mm, the shear stress increased rapidly. This stage was the elastic stage, the shear stress-shear displacement curve was approximately linear and the shear stress increased rapidly with the increase of the shear displacement. After this, the strain hardening stage, the shear stress increased with the increase of shear displacement, but the increasing rate decreased gradually. In the strain hardening stage, the interfacial shear stress between the geomembrane and the solid waste was stable after the shear displacement reached a given value (about 9 cm). The shear stress reached a peak when the shear displacement was within 10–12 mm. At this time, the peak shear stress was the maximum shear strength of the contact interface between geomembrane and desulfurized gypsum under different normal loads.

As can be seen from Figure 4a–r, with the increase of the normal stress, the shear stress at the interface between the geomembrane and each solid waste or soil sample increased significantly. For any industrial solid waste or soil sample, the maximum shear stress of the interface between the rough geomembrane and the solid waste was almost always greater than that of the smooth geomembrane. Under the condition of low normal stress, the maximum shear stress of the interface between the rough and the smooth geomembrane and the solid waste was very close to each other. However, with the increase of the normal stress, the maximum shear stress of the interface between the rough geomembrane and the solid waste was significantly higher than that of the smooth geomembrane. An increasing normal pressure produced a greater difference in maximum shear stress for the rough and smooth geomembranes. This was because the surface friction coefficient of the rough geomembrane was higher; therefore, the friction force between the rough geomembrane and the solid waste was greater. This can be seen more clearly in the following analysis images of maximum shear stress–normal stress curve of contact surface between the smooth and rough geomembranes and different solid wastes or soil samples.

### 3.2. Relationship between the Maximum Shear Stress and the Normal Stress

**Figure 5:**
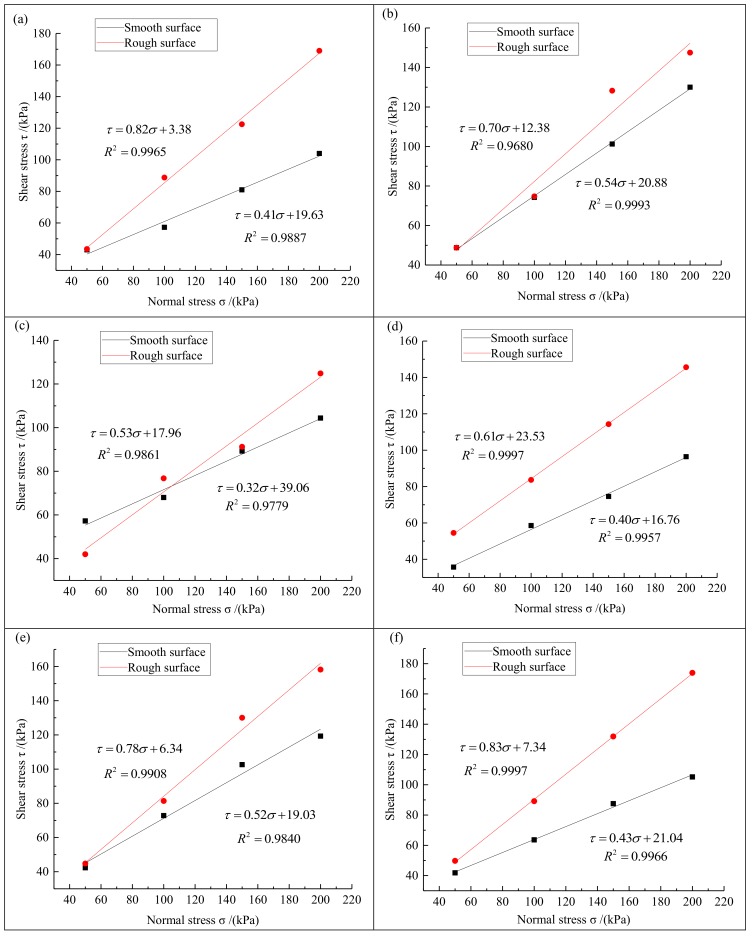

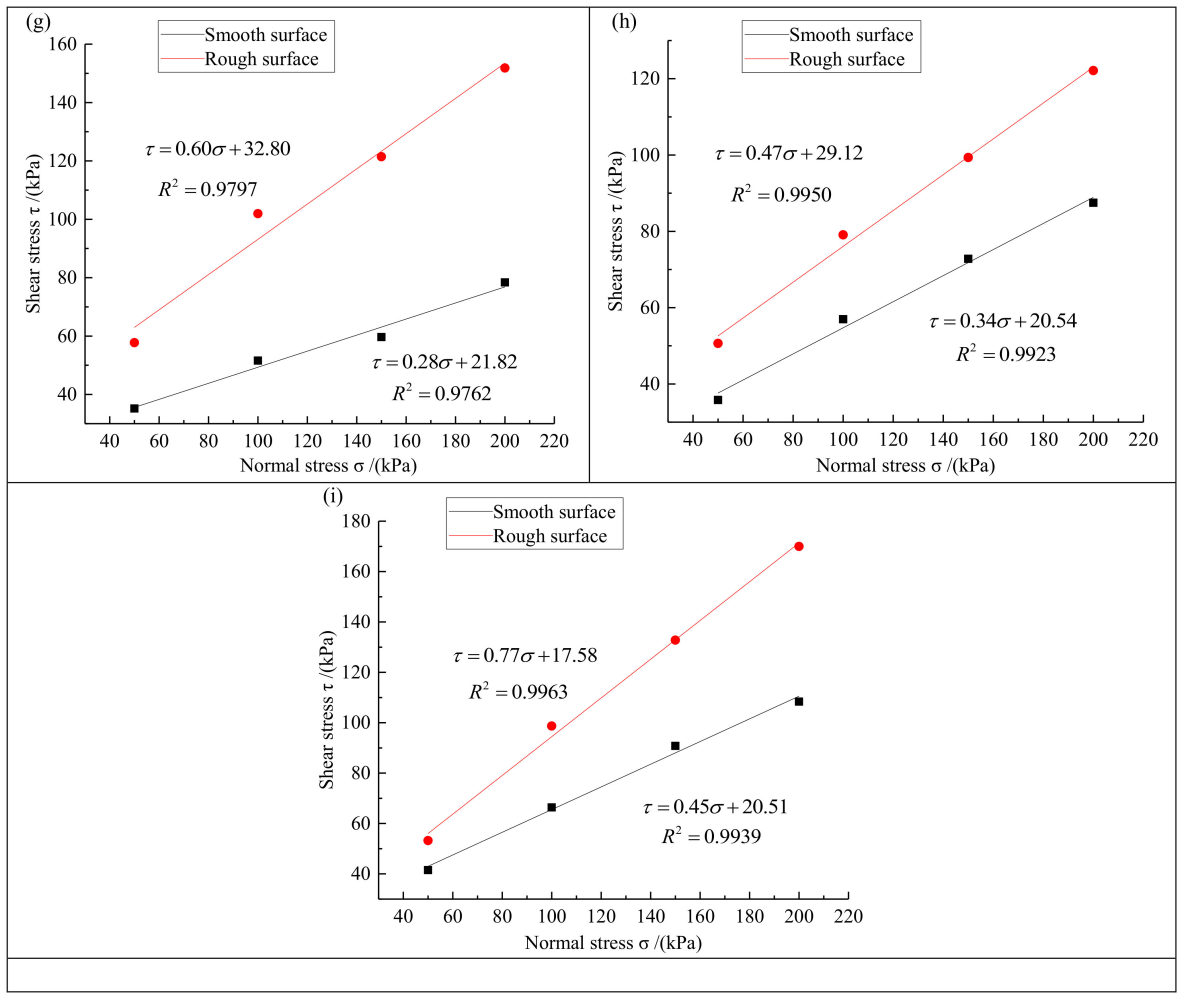
Maximum shear stress–normal stress curve of contact surface between the smooth (black) and rough (red) geomembranes and different solid wastes or soil samples. (**a**) desulfurized gypsum; (**b**) fly ash; (**c**) red mud; (**d**) mercury slag; (**e**) lead-zinc slag; (**f**) manganese slag; (**g**) silica fume; (**h**) clay; (**i**) sand.

The maximum shear stress of the contact interface was obtained from the evolution of the shear stress with the shear displacement and the relationship between the maximum shear stress and the corresponding normal stress was determined. First, four stress points were defined with the normal stress as the abscissa and the corresponding shear stress as the ordinate. Then, the best fit for each point was determined by linear regression. If the error of the fitted straight line was large (the value of the coefficient of determination R^2^ was less than 0.95), then we would carry out the test again until the required result was obtained. The angle between the straight line and the abscissa corresponds to the interfacial friction angle between the geomembrane and the solid waste. The intercept of the straight line on the vertical axis determines the apparent cohesion of the interface between the geomembrane and the solid waste. Figure 5 shows the evolution of the maximum shear stress with the normal stress at the interface between geomembranes with a smooth or rough surface and various solid wastes.

Figure 5 shows that the interfacial shear strength of the geomembrane with a smooth and a rough surface is very close while there is no clear size relationship when the normal stress is small. However, when the normal stress increases gradually, the interfacial shear strength between the geomembrane with a rough surface and any type of solid waste is significantly higher than for the smooth geomembrane. This is because the surface friction coefficient of the rough geomembrane is greater and there is a stronger constraint compaction effect with solid waste particles or soil sample particles. Under the condition of high normal stress (150 kPa and 200 kPa), the lateral friction resistance between the bumps on the surface of the rough geomembrane and the packing particles is more fully played Therefore, the increase of the interfacial shear strength was much higher for the rough geomembrane than for the smooth one for a large normal stress, shear strength and the fracture performance are significantly improved in the rough geomembrane.

Figure 5 shows that the shear strength and normal stress of the interface between smooth geomembrane and rough geomembrane and various solid wastes are linearly related, which conforms to the coulomb shear criterion in soil mechanics. The shear strength formula of the contact interface between geomembrane and solid waste can be expressed as:

(3) $$\tau_{s}=k\times \sigma_{s}+C$$

(4) k=tanφ

where $\tau_{s}$ is the shear strength of the contact interface between geomembrane and solid waste, in kPa, $\sigma_{s}$ is the normal stress applied on solid waste, in kPa, C is the apparent cohesion of the contact interface between geomembrane and solid waste, in kPa, φ is the interface friction angle between geomembrane and solid waste.

Equations (3) and (4) show that the shear strength of the contact interface between geomembrane and solid waste is determined by the friction angle and apparent cohesion. Table 2 shows the friction strength parameters of the contact surface between the geomembrane and the solid waste obtained from Figure 5.

Table 2 shows that the friction angle between all solid wastes or soil samples and the rough geomembrane was larger than for the smooth geomembrane. The apparent cohesion of the interface between the smooth geomembrane and desulfurized gypsum, fly ash, red mud, lead zinc slag, manganese slag and sand is larger than for the rough geomembrane. In contrast, the apparent cohesion of the interface between the smooth geomembrane and mercury slag, silica fume and clay is lower than for the rough geomembrane. The shear strength of the interface between the geomembrane and the solid waste is determined by the friction angle and the apparent cohesion. In our study, the apparent cohesion of the interface between the geomembrane and the solid waste or the soil sample may be affected by various factors, such as the type of solid waste, the particle size and the density. Therefore, there cannot be a regular trend, but the geomembrane with the rough surface was more connected to the various solid waste or soil samples. From the above comparison, it can be found that there is no definite relationship between the apparent cohesion of the contact interface between the smooth geomembrane or rough geomembrane and different solid wastes. And because the apparent cohesion is fixed, it will have a greater impact on the shear strength of the contact interface between geomembrane and solid waste only when the normal stress is small. With the increase of normal stress, the friction angle will have more and more influence on the shear strength of contact interface. The larger the friction angle is, the greater the shear strength of contact interface between geomembrane and solid waste will be. The friction angle of the contact surface was larger for the geomembrane with a rough surface, which is the main reason why its shear strength is higher than that of smooth geomembrane.

### 3.3. Friction Ratio of the Smooth and Rough Geomembranes

To better analyze and compare the friction strength of the contact interface between the smooth and rough geomembrane and different solid wastes under different normal stresses, the friction ratio between the smooth and the rough geomembrane is introduced. It is defined as the ratio of the shear strength (stable value of the shear stress) of the contact interface between the smooth geomembrane and a given solid waste with that for the rough geomembrane for the same normal stress:

(5) $$f=\frac{\tau_{\max}}{\tau_{s,\max}}$$

where $\tau_{\max}$ is the interfacial shear strength of the geomembrane with a smooth surface and $\tau_{s,\max}$ is the interfacial shear strength of the geomembrane with a rough surface under the same normal stress.

**Figure 6:**
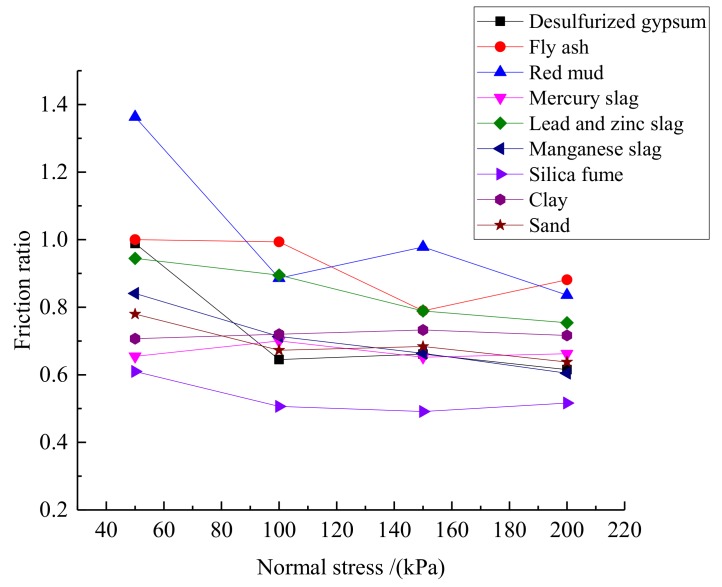
Evolution of the friction ratios for different solid wastes or soil samples.

The friction of the contact interface between various solid wastes or soil samples and the smooth and rough geomembrane was calculated from Equation (5), as shown in Table 3. Figure 6 shows the evolution of the friction ratio for different solid wastes or soil samples.

Table 3 and Figure 6 show that the friction ratio for some of the solid wastes does not have a monotone decreasing trend. For instance, the friction ratio for red mud is 0.886 at a normal stress of 100 kPa and 0.978 at 150 kPa. This is caused by the different compactness of the solid waste during the test. However, the friction ratio for all solid wastes decreases when the normal stress increases. This indicates that a higher normal stress lead to a higher interfacial shear strength in the rough geomembrane than in the smooth one. Additionally, the shear resistance of the rough geomembrane is higher as well. The friction ratio for desulfurization gypsum, red mud and lead-zinc slag is over 0.9 when the normal stress remains small (50 kPa) whereas the friction ratio for mercury slag, silica fume and clay is between 0.6 and 0.7. When the normal stress is high (200 kPa), the friction ratio for almost all solid wastes and soil samples was within 0.6 and 0.8. This means that the friction ratio for various solid wastes decreased gradually when the normal stress increased. When the normal stress was 200 kPa, the friction ratio was significantly smaller than at 50 kPa because the different solid wastes have different particle grades. Under a small normal stress, the solid wastes with a larger particle grade (desulfurized gypsum, red mud and lead-zinc slag) did not make full contact with the geomembrane and the contact area was uneven so that the interfacial friction for the geomembrane with a rough surface could not be fully established. Consequently, the interfacial shear strength was not significantly different for the smooth and rough geomembranes. However, when the normal stress was large, the contact area between the solid waste particles and the geomembrane was more uniform and the shear strength of the geomembrane with a rough surface could fully establish. In terms of mechanical properties of materials, the surface friction coefficient of rough geomembrane is higher than that of smooth geomembrane, so it has a larger interface friction angle. Under the condition of constant apparent cohesion, with the increase of normal stress, the influence of interface friction angle on the shear strength of the interface between geomembrane and solid wastes becomes more and more significant. Therefore, the geomembrane with rough surface has higher shear strength and better shear performance under higher normal load.

## 4. Conclusions

In this work, a series of direct shear tests for the friction failure were carried out on the interface between seven types of main industrial solid waste, two types of soil in the Guizhou Province of China and an HDPE geomembrane. The friction strength of the interface was measured between a smooth and a rough HDPE geomembrane and various solid wastes and The friction strength of the interface was measured between a smooth and a rough HDPE geomembrane and the shear strength characteristics of the interface were analyzed:

(1) When the shear displacement increases, the interfacial shear stress between the HDPE geomembrane and the industrial solid waste did not increase linearly, but parabola. With the increase of shear displacement, the shear stress first increased in a straight line; then the rate of increase gradually decreased. When the shear displacement reached a certain value, the interfacial shear stress remained stable. This means the change of the shear stress between the geomembrane and the solid waste must be carefully considered when designing an anti-seepage structure at a solid waste disposal site.
(2) The interfacial shear strength between the geomembrane with a rough surface and the solid waste was close to that for the smooth geomembrane for a small normal stress. The interfacial shear strength between the rough geomembrane and the solid waste was significantly higher than for the smooth geomembrane for larger normal stresses. This is because when the normal stress was large, the solid waste particles was in closer contact with the surface of the geomembrane; the lateral friction resistance between the bumps on the surface of the rough geomembrane and the solid waste particles was more fully developed.
(3) The shear strength of the interface between geomembrane and solid waste soil was determined by the friction angle and apparent cohesion. With the increase of the normal stress, the shear strength of the interface was mainly determined by the interface friction angle. The interface friction angle of rough geomembrane was higher than that of smooth geomembrane, therefore, the geomembrane with a rough surface had a better shear resistance and a better tensile crack resistance.

## Figures and Tables

**Table 1:** Physical properties of HDPE geomembranes (minimum).

Type	Thickness/mm	Density/(g/cm^3^)	Yield Strength/(N/mm)	Yield Elongation/%	Fracture Strength /(N/mm)	Elongation at Break /%	Right-Angled Tearing Strength/N	Puncture Strength /N
Smooth	2.5	0.939	37	12	67	700	311	800
Rough	2.5	0.939	37	12	26	100	311	667

**Table 2:** Experimental results of direct shear friction characteristics of geomembranes.

Type	Interface	Friction Angle/(°)	Apparent Cohesion/(kPa)
Desulfurized gypsum	Smooth	22.29	19.63
	Rough	39.35	3.38
Fly ash	Smooth	28.37	20.88
	Rough	34.99	12.38
Red mud	Smooth	17.74	39.06
	Rough	27.92	17.96
Mercury slag	Smooth	21.8	16.76
	Rough	31.38	23.63
Lead-zinc slag	Smooth	27.47	19.03
	Rough	37.95	6.34
Manganese slag	Smooth	23.27	21.04
	Rough	39.69	7.34
Silica fume	Smooth	15.64	21.82
	Rough	30.96	32.8
Clay	Smooth	18.78	20.54
	Rough	25.17	29.12
Sand	Smooth	24.23	20.51
	Rough	37.56	17.58

**Table 3:** Friction ratio for each solid waste or soil sample for different normal stresses.

Type/Normal Stress	50 kPa	100 kPa	150 kPa	200 kPa
Desulfurized gypsum	0.989	0.645	0.661	0.615
Fly ash	1.000	0.993	0.789	0.881
Red mud	1.363	0.886	0.978	0.836
Mercury slag	0.655	0.700	0.652	0.662
Lead–zinc slag	0.945	0.895	0.789	0.754
Manganese slag	0.841	0.714	0.664	0.605
Silica fume	0.610	0.506	0.491	0.516
Clay	0.707	0.720	0.733	0.716
Sand	0.780	0.673	0.684	0.638

## References

[1] Rowe R.K., Sangam H.P. (2002). Durability of HDPE Geomembranes. Geotext. Geomembr..

[2] Xue Q., Zhang Q., Li Z.Z., Xiao K. (2013). The Tension and Puncture Properties of HDPE Geomembrane under the Corrosion of Leachate. Materials.

[3] Liu N.E., Ramsey B. (2017). Leak Location Using Permanent Leak Detection System for HDPE Geomembrane. Chin. J. Chem. Eng..

[4] Cen W.J., Bauer E., Wen L.S., Wang H., Sun Y.J. (2019). Experimental Investigations and Constitutive Modeling of Cyclic Interface Shearing between HDPE Geomembrane and Sandy Gravel. Geotext. Geomembr..

[5] Power C., Ramasamy M., Macaskill D., Shea J., MacPhee J., Mayich D., Baechler F., Mkandawire M. (2017). Five-Year Performance Monitoring of a High-Density Polyethylene (HDPE) Cover System at a Reclaimed Mine Waste Rock Pile in the Sydney Coalfield (Nova Scotia, Canada). Environ. Sci. Pollut. R.

[6] Eldesouky H.M.G., Brachman R.W.I. (2019). Viscoplastic Modelling of HDPE Geomembrane Local Stresses and Strains. Geotext. Geomembr..

[7] Negussey D., Wijewickreme W.K.D., Vaid Y.P. (1989). Geomembrane Interface Friction. Can. Geotech. J..

[8] Chen Z., Gong H., Zhang M., Weili W. (2011). Impact of Using High-Density Polyethylene Geomembrane Layer as Landfill Intermediate Cover on Landfill Gas Extraction. Waste Manag..

[9] Rowe R.K., Islam M.Z., Hsuan Y.G. (2010). Effects of Thickness on the Aging of HDPE Geomembranes. J. Geotech. Geoenviron..

[10] Abdelaal F.B., Rowe R.K., Islam M.Z. (2014). Effect of Leachate Composition on the Long-Term Performance of a HDPE Geomembrane. Geotext. Geomembr..

[11] Saheli P.T., Rowe R.K., Petersen E.J., O’Carrol D.M. (2017). Diffusion of Multiwall Carbon Nanotubes through a High-Density Polyethylene Geomembrane. Geosynth. Int..

[12] Department of Environmental Protection of Guizhou Province (2013). Standard for Pollution Control on the Storage and Disposal Site Forgeneral Industrial Solid Wastes in GuiZhou Province DB52/865-2013.

[13] Cheng B.G., Guo C.J., He X.Y., Fu J. (2018). Application of Composite Geomembranes in Seepage Protection for Changhe Dam Hydropower Station Cofferdam. Heilongjiang Hydraul. Sci. Technol..

[14] Liu W.N. (2010). On Application of Composite Geo-Membrane in Dike’s Anti-Seepage of the Channel High Filled Section. Shanxi Archit..

[15] Zhou J.C. (2003). Application of HDPE Geomembrane in Municipal Solid Waste Landfill. Adv. Sci. Technol. Water Resour..

[16] Liu S.L. (2015). Anti-Seepage Technology of HDPE Geomembrane in Landfill. Resour. Econ. Environ. Prot..

[17] Li Y., Song X.G., Luan J.L. (2014). Research on Mechanism of Geomembrane/Geotextile Interface Strength. J. Water Resour. Archit. Eng..

[18] Tian K., Benson C.H., Yang Y., James M.T. (2018). Radiation Dose and Antioxidant Depletion in a HDPE Geomembrane. Geotext. Geomembr..

[19] Rowe R.K., Islam M.Z. (2009). Impact of Landfill Liner Time-Temperature History on the Service Life of HDPE Geomembranes. Waste Manag..

[20] Eithe A.W., Koerner G.R. (1997). Assessment of HDPE Geomembrane Performance in a Municipal Waste Landfill Double Liner System After Eight Years of Service. Geotext. Geomembr..

[21] Sangam H.P., Rowe R.K. (2002). Effects of Exposure Conditions on the Depletion of Antioxidants From High-Density Polyethylene (HDPE) Geomembranes. Can. Geotech. J..

[22] Ling H.I., Pamuk A., Dechasakulsom M., Mohri Y., Burke C. (2001). Interactions between PVC Geomembranes and Compacted Clays. J. Geotech. Geoenviron. Eng..

[23] Fox P.J., Ross J.D., Sura J.M., Thiel R.S. (2011). Geomembrane Damage Due to Static and Cyclic Shearing over Compacted Gravelly Sand. Geosynth. Int..

[24] Lin W.A., Zhang H.W., Zhan L.T., Chen Y.M. (2012). Large-Scale Ramp Model Tests on Geomembrane/Geotextile Interface. Chin. J. Geotech. Eng..

[25] Gao J.L., Zhang M.X., Zhang W.J. (2011). Interface Property between Sand and Reinforced Geomembrane. Rock Soil Mech..

[26] Zhu S.R., Xu C., Ding J.H. (2018). Laminated Shear Test of Geotextile-Sand Interface. Rock Soil Mech..

[27] Zhang C., Zhu Z.D., Zhu S. (2019). Nonlinear Creep Damage Constitutive Model of Concrete Based on Fractional Calculus Theory. Materials.

[28] Ferreira F., Vieira C., Maria D.L.L. (2016). Cyclic and Post-cyclic Shear Behaviour of a Granite Residual Soil—Geogrid Interface. Procedia Eng..

[29] Basudhar P.K. (2010). Modeling of Soil–Woven Geotextile Interface Behavior from Direct Shear Test Results. Geotext. Geomembr..

[30] Zhu Z.D., Zhang C. (2019). A Statistical Damage Constitutive Model Based on the Weibull Distribution for Alkali-Resistant Glass Fiber Reinforced Concrete. Materials.

[31] Frost J.D., Kim D., Lee S.W. (2012). Microscale geomembrane-granular material interactions. KSCE J. Civil Eng..

[32] Cen W.J., Wang H., Sun Y.J., Wen L.S. (2018). Monotonic and Cyclic Shear Behaviour of Geomembrane-Sand Interface. Geosynth. Int..

[33] Bacas B.M., Cañizal J., Konietzky H. (2015). Shear Strength Behavior of Geotextile/Geomembrane Interfaces. J. Rock Mech. Geotech. Eng..

[34] Standardization Research Institute of China Textile General Association (1998). Geotextiles and Geotextile-Related Products-Determination of Friction Characteristics GB/T 17635.1-1998.

